# Occurrence and Risk Assessment of Dietary Exposure to Deoxynivalenol in Wheat-Based Products Based Different Wheat-Producing Area for the Inhabitants in Shanghai, China

**DOI:** 10.3390/jof7121015

**Published:** 2021-11-26

**Authors:** Xianli Yang, Zhiyong Zhao, Jianhua Wang, Junhua Yang, Hengchao E, Bo Chen, Pengzhen He, Yanglan Tan, Changyan Zhou

**Affiliations:** 1 Laboratory of Quality & Safety Risk Assessment for Agro-Products (Shanghai), Institute for Agro-Food Standards and Testing Technology, Ministry of Agriculture, Shanghai Academy of Agricultural Sciences, Shanghai 201403, China; zhaozhiyong@saas.sh.cn (Z.Z.); wangjianhua@saas.sh.cn (J.W.); yangjunhua@saas.sh.cn (J.Y.); ehengchao@saas.sh.cn (H.E.); 2 Shanghai Co-Elite Agro-Food Testing Technical Service Co., Ltd., Shanghai 201106, China; 3 Department of Nutrition and Food Hygiene, School of Public Health, Fudan University, Shanghai 200433, China; chenb@fudan.edu.cn; 4 College of Chemistry and Chemical Engineering, Mudanjiang Normal University, Mudanjiang 157012, China; hepengzhenadu@163.com; 5 CAS Key Laboratory of Nutrition, Metabolism and Food Safety, Shanghai Institute of Nutrition and Health, Chinese Academy of Sciences, Shanghai 200031, China; yltan@sibs.ac.cn

**Keywords:** risk assessment, deoxynivalenol, wheat-based products, wheat production area, inhabitants in Shanghai

## Abstract

Deoxynivalenol (DON) is one of the major mycotoxins that contaminate cereals. In this study, we determined the DON level in wheat-based products from Chinese five main production areas collected in Shanghai and calculated the daily intake of DON for inhabitants using the point evaluation and the probabilistic evaluation based on Monte Carlo simulation. The results showed the positive rates of DON in the products were higher than 80.0%, with the concentrations ranging from 41.8 to 1110 µg/kg. The estimated mean daily intakes of DON for 7- to 10-year-old children and adults groups were below 1 µg/kg bw/day, the provisional maximum tolerable daily intake (PMTDI) set by the Joint FAO/WHO Expert Committee on Food Additives (JECFA), suggesting no health risks for the consumers. However, the 99th percentiles of dietary DON exposures for children and adults exceeded the PMTDI, indicating adverse health effects might occur if the two groups intake highly contaminated wheat-based products. The potential health risks for the two groups exposed to DON in the wheat-based products from the Middle and Lower Yangtze Valley (MLYV) were higher than those from the other areas in China.

## 1. Introduction

Deoxynivalenol (DON), one of the most important type B trichothecenes [1], is toxic and has adverse health effects on humans and animals [2]. The toxin is often detected worldwide in cereals and cereal-based products, including wheat and maize [3], especially in humid and rainy, temperate areas [4,5].

DON occurrence in cereals has received increasing attention over the past few decades [6,7]. According to a study by Golge et al. [8], the positivity rate of DON, the most prevalent mycotoxin, in cereals and derived products collected in Turkey from 2015 to 2018 was 26%. The contaminated incidence of DON in maize from Zimbabwe was 24%, with the mean concentration of 217 µg/kg [9]. During 2008–2011, a total of 697 samples of wheat and wheat-based products in China were analyzed for multiple mycotoxins. The results showed that DON was the most frequently found toxin [10]. In particular, the DON concentration in nine wheat flour samples exceeded the Chinese regulatory limit of 1000 μg/kg recommended by the National Health and Family Planning Commission of China (NHFPC) [11]. According to a report, DON is the most abundant toxin detected in wheat samples in part of China [12]. In the study, the DON-positive rate was 90.8% (307/338) with average concentration 2846.7 μg/kg (range from 2 μg/kg to 59,278.0 μg/kg), and the level of DON in 44.7% (151/338) samples exceeded the maximum tolerance limit of 1000 μg/kg regulated by Chinese government. Therefore, risk assessment of DON in food is necessary to guarantee consumer health.

Risk and hazard assessment of dietary exposure to DON in cereals and derivative products has been carried out [9,13,14]. Using the assessment methods for risk and hazard of environmental pollutants, assessment methods for substances in food have also been developed. Monte Carlo probabilistic simulation is often employed to predict the cumulative risk of DON in food. Sun et al. [15] assessed the dietary exposure to DON from cereals in China during the period 2010–2013 using Monte Carlo probabilistic simulations. Their results indicated that 75% of children and 90% of the general population and the adults were below the provisional maximum tolerable daily intake (PMTDI) of 1 µg/kg bw/day set by the Joint FAO/WHO Expert Committee on Food Additives (JECFA) [16]. According to a risk assessment in Catalonia, Spain, DON could be detected in human urine from people who ingest bread, pasta, and pastries [17]. In Netherlands, the dietary intake of DON in wheat exceeded a provisional tolerable daily intake (PTDI) of 1.1 µg/kg bw/day during September 1998 to January 2000. Moreover, 80% of the 1-year-old children had a DON intake above the PTDI [18]. Nielsen et al. found fetuses can be exposed to DON during pregnancy, which may affect mothers and fetuses’ health [19].

**Figure 1:**
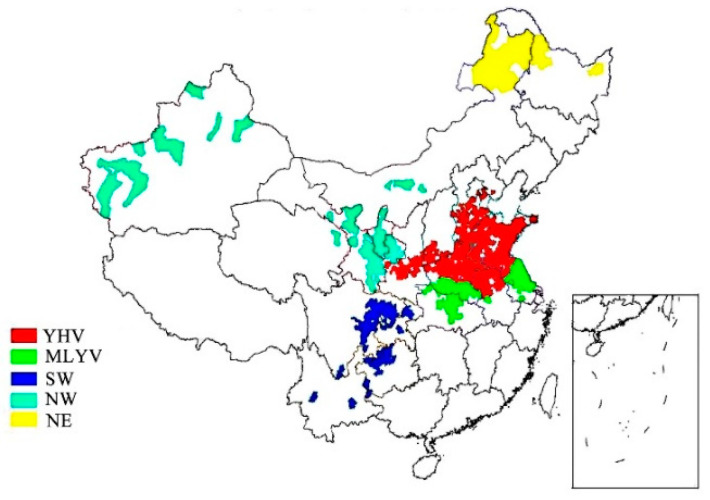
Sketch map of the five main wheat production areas in China (YHV, the Yellow and Huai Valley; MLYV, the Middle and Lower Yangtze Valley; SW, the southwest area; NW, the northwest area; NE, the northeast area) [20].

In China, the main wheat production areas, such as the Middle and Lower Yangtze Valley (MLYV), the Yellow and Huai Valley (YHV), the northeast area (NE), the northwest area (NW), and the southwest area (SW), are located in the wet northern temperate zone, shown in Figure 1 [20]. Therefore, DON is frequently found in China [21,22,23], especially in rainy years. In Shanghai, the inhabitants consume about 6.34 million tons of grains every year, 80% of which is supplied from other geographical areas [24]. Quality levels of cereal products and the impact on consumer health are different for cereal from different areas. The risk assessment for the dietary intake of DON in cereal-based products from different geographical has not been reported. Nevertheless, a previous study has evaluated the dietary exposure to DON in cereals and cereal-based products for adults and children in Shanghai [14].

The objective of this study is to: (1) analyze the occurrence of DON in wheat-based products from Chinese five main production areas collected in Shanghai and (2) evaluate the potential health risk of exposure to DON by point evaluation and probabilistic evaluation for inhabitants in Shanghai.

## 2. Materials and Methods

### 2.1. Toxins and Chemicals

DON standard solution, ammonium acetate, and formic acid were purchased from Sigma-Aldrich (St. Louis, MO, USA) and stored at −20 °C until use. The standard solution was diluted in acetonitrile prior to analysis to obtain the working solution. HPLC-grade acetonitrile and methanol were obtained from Merck (Darmstadt, Germany). Ultrapure water was produced by a Milli-Q filtration system (Bedford, MA, USA). All of the other reagents were of HPLC or analytical grade.

### 2.2. Samples

A total of 340 samples of wheat-based products were randomly collected in Shanghai markets, including supermarkets, chain stores, and farm product markets, in all 16 city districts. Information concerning the geographic origin of the samples was recorded. All of the samples were ground into fine powder and stored at −20 °C under dry and dark conditions before analysis.

### 2.3. Determination of DON Concentration in Wheat-Based Products

The wheat-based products were pretreated according to the standard operating procedures set by the NHFPC [25]. Of each sample, 25 g was mixed in 100 mL water. The mixture was shaken for 20 min and centrifuged at 6000 rpm for 10 min. The supernatant was collected and centrifuged at 10,000 rpm for 5 min. The supernatant was passed through DonStar immunoaffinity column (3 mL of antibody gel, Romer Labs, Inc. Union, MO, USA) at a rate of 2 mL/min, and then 5 mL of PBS and 5 mL of water were passed through the column at a flow rate of 1–2 drops per second. The columns were eluted with 2 mL methanol at a rate of 1 drop per second and then dried by nitrogen gas flow at 50 °C. The residues were reconstituted in 1 mL methanol/water (20/80, *v*/*v*) solution and passed through a 0.22-μm nylon filter (Millipore Corp., Billerica, MA, USA). The filtrate was collected for UPLC-MS/MS analysis.

Sample analysis was performed using an Acquity ultra high-performance liquid chromatography (UHPLC) system (Waters, Eschborn, Germany) coupled to a triple quadrupole mass spectrometer 5500 (AB Sciex, Darmstadt, Germany) equipped with an electrospray ionization source operating in the positive mode. Chromatographic separation was carried out on an Acquity BEH C18 column (2.1 mm × 100 mm, 1.8 μm; Waters) at the temperature of 40 °C. The mobile phase consisted of water containing 5 mmol/L ammonium acetate (solvent A) and methanol (solvent B). A linear gradient program was applied at a flow rate of 300 µL/min as follows: 0–1 min, 90% A; 1–5 min, 10% A; 5–6 min, 10% A; 6–6.5 min, 90% A; and 6.5–8 min, 90% A. The sample temperature was set at 4 °C, and the sample injection volume was 5 μL. The operating parameters of AB Sciex 5500 were set as ion spray voltage, 5500 V; interface heater temperature, 450 °C; curtain gas, 40 psi; ion source gas 1, 50 psi; ion source gas 2, 50 psi; and collision activated dissociation (CAD) gas, medium. Multiple reactions monitoring (MRM) mode was employed for quantitative analysis. DON was monitored using the followed fragmentation pathways: m/z 297.3 → m/z 203 (quantifier signal), m/z 297.3 → m/z 175.1 (confirmation signal). Data acquisition and processing were performed using Analyst 1.6.2 software (AB Sciex). The recovery of the method was 82.3–95.2% in the spiked wheat samples. The DON limit of detection (LOD) of the standard method is 5 μg/kg, and the limit of quantitation (LOQ) of the standard method is 15 μg/kg.

### 2.4. Dietary Consumption Data

The data on food consumption of children were obtained from a Shanghai Food Consumption Survey (SHFCS) performed by Fudan University between 2012 and 2014. The detail of the SHFCS was described in previous studies [14,26]. In this study, the survey collected the food consumption data of 125 (65 boys and 60 girls) 7- to 10-year-old children and 1269 (aged 17–60 years, 608 adult men and 661 adult women) participants residing in Shanghai. A face-to-face questionnaire survey was conducted to record the following information: food types, amount and frequency of eating, 24-h dietary recall, and real-time measurement of body weight.

### 2.5. Risk Assessment

Two mathematical approaches, i.e., point evaluation and probability evaluation, were employed to assess the health risk resulting from DON intake through wheat-based products. The point evaluation was performed by multiplying the amount of food consumption (mean or percentile) with the DON concentration in each sample. The corresponding equations are the following [27]:

*y* = (*x* ∙ *c*)/*w* (1)

MOS = *y*/TDI (2)

where, *y* is the daily exposure level of an individual to a specific substance (μg/kg bw/day), *x* is the mean amount of food consumption (g/d), *c* is the content of a substance in food, and *w* is the mean body weight (kg). The MOS (Equation (2)) is calculated by dividing the daily exposure level by the TDI, which is defined as the maximum amount of one substance that can be ingested daily over a lifetime with no appreciable health risk. MOS values lower than 1 are generally considered to indicate no risk to human health. If the MOS value is greater than 1, the exposure risk of one specific substance is undesirable.

The uncertainty caused by the small size of each sample study and unreliability of food consumption data can result in inaccurate risk analysis. The Monte Carlo model is used to quantify and decrease the uncertainty in the health risk assessment. In our study, the cumulative exposure of each substance was performed using the Monte Carlo model operated in @RISK Version 5.0 (Palisade, Ithaca, NY, USA) in combination with Microsoft Excel 2016. The daily food consumption amounts and the sample residue concentration data were input into @RISK software and integrated by Monte Carlo sampling with a number of 5000 iterations.

## 3. Results and Discussion

### 3.1. The Origin of Wheat-Based Products Sold in Shanghai

**Figure 2:**
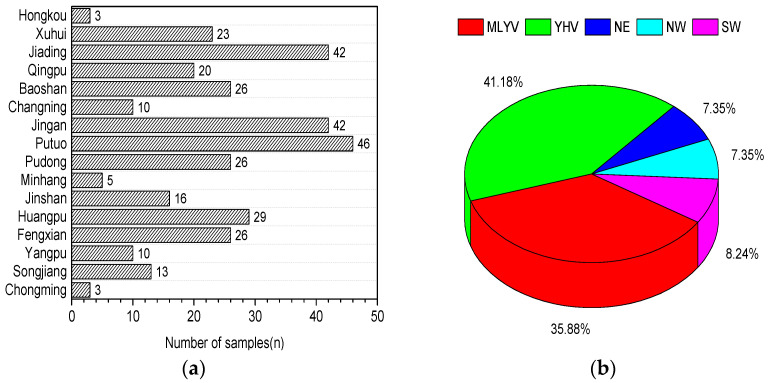
(**a**) The number of wheat-based products samples and the sample collection location in Shanghai city; (**b**) the proportion of the geographic origin of the samples.

In the present study, 340 wheat-based products were randomly collected from Shanghai markets, including supermarkets, chain stores, and farm product markets, in all 16 city districts. The number of samples from every district is shown in Figure 2a. The wheat samples in Shanghai mainly originates from MLYV (35.9%), YHV (41.2%), NE (7.35%), NW (7.35%), and SW (2.9%), as shown in Figure 2b. The wheat samples from MLYV and YHV together represent 77.1% of the total. This is not surprising, as these are currently the main wheat-producing areas in China [28].

### 3.2. Dietary Intake of Children and Adults in Shanghai

In Shanghai, the daily diets of 125 seven- to 10-year-old children (65 boys and 60 girls) and 1269 adults (608 adult men and 661 adult women) were surveyed, as shown in Table 1. The mean and median daily intake of wheat flour products, 98.7, 67.0 g/d for boys and 75.1, 57.9 g/d for girls, were lower than the mean consumption of wheat products for teenagers (129.6 g/d) in Parana, Brazil [29]. However, the 90th percentile (230.0, 181.1 g/d), 95th percentile (303.4, 257.9 g/d), 97.5th percentile (361.5, 285.5 g/d), and 99th percentile (513.6, 309.4 g/d) of daily intake for seven- to 10-year-old children were higher than the value. Similarly, the mean and median daily intake of wheat flour products of 95.5, 67.0 g/d for adult men and 72.2, 40.0 g/d for adult women were lower than the mean consumption of wheat products for adults (117.6 g/d). The 90th percentile (250.0, 181.1 g/d), 95th percentile (303.4, 257.9 g/d), 97.5th percentile (361.5, 285.5 g/d), and 99th percentile (513.6, 309.4 g/d) of daily intake for seven- to 10-year-old children exceeded the mean consumption of wheat products for adults in Parana, Brazil. The 97.5th and 99th percentiles of daily intake of wheat flour products for children and adults were also higher than the average consumption for adults (267.7 g/d) in Serbia [30].

### 3.3. Contamination Levels of Wheat Flour Products

Of the 340 collected samples of wheat flour products, 288 samples (84.7%) were DON positive, as shown in Table 2. The mean, median, and 90th, 95th, 97.5th, and 99th percentiles of DON content in products with wheat from different areas are also shown in Table 2. The positivity rate of DON in each area was higher than 80.0%. The positivity rate of DON in NE was 100% (25/25). The mean DON content in MLYV, YHV, and total samples was 288.2 ± 269.6 µg/kg (ranging from 41.8 to 1110 μg/kg), 148.7 ± 188.8 µg/kg (ranging from 42.0 to 896 μg/kg), and 205.6 ± 234.7 µg/kg, respectively. The values of the other areas were lower. Moreover, the 99th percentiles of DON content in MLYV, YHV, and total samples were 972.9 µg/kg, 886.3 µg/kg, and 934.2 µg/kg, respectively, i.e., all three slightly below the maximum limit of 1000 µg/kg in raw wheat and derived foods [11] as determined by the Codex Alimentarius Commission (CAC) [31]. However, the 95th percentile of DON content in MLYV and total samples and the 97.5th percentile in YHV exceeded the maximum limits of 750 and 500 µg/kg established by the European Commission (EC) [32]. Areas with higher humidity showed higher DON levels in the wheat samples [33].

The results, a DON positivity rate of 86.7% and a mean concentration of 325 ± 293 μg/kg (range from 17.5 to 976 μg/kg), were comparable with results from Serbia [34], higher than the reported DON positivity rate of 43.2% and mean concentration of 18.8 µg/kg (range from 3.1 to 172.9 µg/kg) in 188 cereal samples (consisting of rice, brown rice, barley, and maize) in South Korea [35], and lower than the DON positivity rate of 97.2% and mean concentration of 591 µg/kg (range from 60 to 1720 µg/kg) in biscuits from Brazil [36]. In addition, the results were lower than the values reported in 2013 (positivity rate of 98.7%, mean concentration of 879.3 ± 1127.8 μg/kg), 2014 (positivity rate of 84.7%, mean concentration of 627.8 ± 640.5 μg/kg), and 2015 (positivity rate of 98.7%, mean concentration of 1628.6 ± 2168.0 μg/kg) from wheat grown in Jiangsu, China [37].

As well known, temperature and rainfall are the key climatic factors having a great impact on the occurrence of wheat Fusarium head blight (FHB) as well as the contamination level of mycotoxins [38,39]. The climate conditions in the two wheat-producing areas (MLYV and YHV) are humid and warm during wheat cultivation, especially at the flowering and grain filling stages [4,40], which are contributed to Fusarium infection and DON synthesis [41]. This is why the average concentration of DON in wheat produced in MLYV is higher than that in NE, NW, and SW. The results are comparable to previously published studies, in which DON levels of wheat samples collected from Northwestern of China are lower than those in samples collected in the Yangtze-Huaihe river basin region [4,42].

### 3.4. Point Evaluation of DON Dietary Exposure

Based on the DON content in wheat flour and the dietary intake, dietary DON exposure through wheat-based products from different areas was calculated for seven- to 10-year-old children and adults from Shanghai. The results are shown in Table 3, Table 4, Table 5 and Table 6.

The mean dietary DON exposure of 0.530 µg/kg bw/day (i.e., 53.0% of the PMTDI value of 1 μg/kg bw/day [43]) for 7- to 10-year-old boys in total samples was higher than in all of the areas except MLYV (0.743 µg/kg bw/day). The median dietary exposure in each area for the four groups was below the PMTDI value. In addition, the 90th percentiles of dietary exposures from NE for adults, the 90th and 95th percentiles of dietary exposures from NW for children, and the 90th, 95th and 97.5th percentiles of dietary exposures for adults from NW were below 1 μg/kg bw/day. These results indicate that there are no increased health risks for most seven- to 10-year-old children and adults caused by recent DON exposure through the wheat-based products from the above areas.

However, the 90th percentiles of dietary exposure for boys from MLYV (4.55 µg/kg bw/day), YHV (2.11 µg/kg bw/day), NE (1.42 µg/kg bw/day), and total samples (3.01 µg/kg bw/day) were all above the PMTDI. The 97.5th percentiles from NW and SW for seven- to 10-year-old girls were 1.19 and 1.10 µg/kg bw/day, respectively, also exceeding the PMTDI. For girls, the 90th percentiles of dietary exposure from MLYV, YHV, NE, and total samples were 4.10, 1.90, 1.28, and 2.71 µg/kg bw/day, respectively, which were all above the PMTDI as well. Additionally, the 97.5th percentile of DON exposure from NW (1.08 µg/kg bw/day) and the 99th percentile from SW (1.10 µg/kg bw/day) were higher than the PMTDI. Similarly, the 90th, 95th, 97.5th and 99th percentiles of dietary exposures for adults from MLYV, YHV, total samples, the 95th, 97.5th, and 99th percentiles of dietary exposures for adults from NE and 99th percentiles of dietary exposures for adults from NW were above the PMTDI. The results indicate that extreme DON exposure values do pose risks. The risk of DON intake through products from MLYV is higher than that from the four other areas. Seven- to 10-year-old children showed higher levels of dietary DON intake than adults.

Our results are consistent with previous reports. The estimated daily DON intake from cereal-based baby food for four-, five-, six-, and seven- to 12-month-old infants (*n* = 60) in Cantabria and Aragón, Spain [44], were 0.25, 0.28, 0.32, and 0.36 µg/kg bw/day, respectively, lower than the tolerable daily intake (TDI) of 1 µg/kg bw/day established by the European Food Safety Authority (EFSA) [45].The results were higher than the dietary DON exposure of children and of adults through cereal-based foods (*n* = 159) of 0.05 µg/kg bw/day and 0.006 µg/kg bw/day in Valencia, Spain [46].Those results indicated the consumers had no increased health risks. However, the daily DON intakes of adolescents in Belgium [47], two- to six-year-old children in Australia [47], and children in Serbia [35] of 1.26, 3.16, and 1.7 μg/kg bw/day, respectively, were higher than the PMTDI. Moreover, the daily DON intakes of adults in Brazil [47] (1.45 μg/kg bw/day) and Belgium [47] (1.08 μg/kg bw/day) were higher than the PMTDI of 1 μg/kg bw/day, suggesting an increased health risk is associated with wheat product consumption.

Compared with other studies in China, the mean DON exposure through wheat-based products in our study is lower than that from wheat flour products for Chinese children (3.9 µg/kg bw/day) and adults (1.3 µg/kg bw/day) [48]. Wang et al. [49] reported the 90th percentile of exposure to DON through wheat flour- and corn-based products collected from Chinese supermarkets and farmers markets for three- to 13-year-old children (2.066 µg/kg bw/day) and for ≥14-year-old (1.28 µg/kg bw/day), higher than PMTDI, indicating the consumer were at high risk. Sun et al. [15] found the 90th percentile of estimated dietary DON intake through cereals and cereal-based foods for two- to six-year-old children was 1.2 µg/kg bw/day, 120% of the PMTDI, and the 99th percentile of estimated dietary DON intake through cereals and cereal-based foods for adults was 3.0 µg/kg bw/day, three times the PMTDI, indicating the consumption of wheat flour- and wheat-based products poses a potential health risk in China. Deng et al. [50] estimated the mean daily DON intake for two- to 12-year-old children (2.09 µg/kg bw/day), for 13- to 18-year-old adolescents (3.08 µg/kg bw/day), and for 18- to 65-year-old adults (1.41 µg/kg bw/day) in Henan, China, and reported values exceeding the PMTDI. Similarly, the mean and the 90th percentile of estimated dietary DON intake for seven- to 12-year-old children in Henan, China, were 3.80 and 9.93 µg/kg bw/day, respectively, 3.80 and 9.93 times the PMTDI [51]. Wang et al. [52] found a mean DON exposure through wheat-based products for seven- to 12-year-old children, for 13- to 17-year-old adolescents, and for 18- to 59-year-old adults of 3.2, 2.5, and 2.4 µg/kg bw/day in Anhui, China, which is 3.2, 2.5, and 2.4 times the PMTDI, respectively. In contrast, Liu et al. [21] found mean levels of daily DON intake through wheat products in Hebei province, China, for adults of 0.49 µg/kg bw/day in 2011, 0.86 µg/kg bw/day in 2012, and 0.56 µg/kg bw/day in 2013, which were all below the PMTDI. Also, the mean and median dietary DON intake (as calculated from urinary values) for seven- to 12-year-old children and 18- to 59-year-old adults in Sichuan, China, were reported to be 0.60 and 0.41 µg/kg bw/day, respectively, less than or equal to the PMTDI, indicating there is no health risk for this age group [51].

In addition, it is worth noting the areas where higher DON intake was the previously reported, such as Henan and Anhui, lie in MLYV and YHV, and the lower-intake areas, such as Hebei and Sichuan, lie in the other areas. In MLYV and YHV, abundant rainfall and warm temperatures [53], provides a superior environment for *F. asiaticum*, one of its metabolites is DON. Our results on DON intake through consumption of wheat from different areas are comparable to previous results.

### 3.5. Probabilistic Evaluation of Dietary DON Exposure

**Figure 3:**
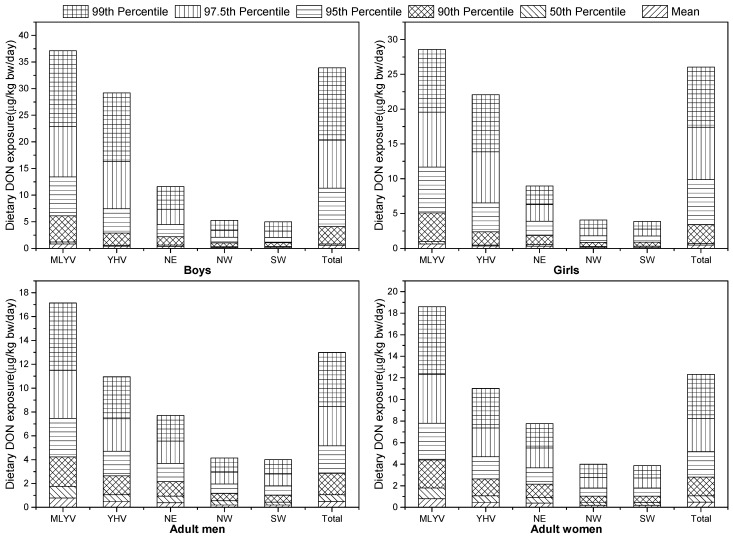
The estimated dietary DON exposure through wheat-based products from different production areas for 7- to 10-year-old children and adults determined by probabilistic evaluation.

Point evaluation, which does not consider the variability and uncertainty of food consumption and contamination levels, might give an overevaluation of the exposure levels [27]. Therefore, a full probabilistic method, the Monte Carlo model, was used for further investigation to provide more realistic estimated DON exposures through wheat from different wheat producing areas for seven- to 10-year-old children and adults, as shown in Figure 3.

The mean and median probabilistic daily DON intake from each area were below the PMTDI, meaning that the margin of safety (MOS) values were less than 1 and that there was no increased health risk for seven- to 10-year-old children and adults due to DON intake through wheat products. The mean probabilistic DON exposure values were similar to the point evaluation values, which were comparable to previous reports from different countries. Assunção et al. [54] found that the mean probabilistic dietary DON exposure through cereal-based products for Portuguese children (*n* = 52) was 0.0539 µg/kg bw/day, 5.39% of the PMTDI. Additionally, the probabilistic estimated daily DON intake through wheat-based products for adults in Croatia (0.121 μg/kg bw/day), Greece (0.181 μg/kg bw/day), and Serbia (0.262 μg/kg bw/day) was below the PMTDI [55]. Ortiz et al. [56] used first-order Monte Carlo simulation based on 10,000 iterations and estimated a daily DON exposure through wheat noodles for children aged 0–23 months (*n* = 128) in Ecuadorian highlands of 0.0915 μg/kg bw/day.

However, the 90th percentile of daily DON intake through wheat-based products from MLYV, YHV, NE, and total samples, the 95th percentile from NW, and the 97.5th percentile from SW for seven- to 10-year-old boys were higher than the PMTDI. Moreover, the 90th percentile of daily DON intake through wheat-based products from MLYV, YHV, NE, and total samples, the 97.5th percentile from NW, and the 99th percentile from SW for seven- to 10-year-old girls were higher than the PMTDI. Similarly, the 90th percentile of daily DON intake through wheat-based products from MLYV, YHV, NE, and total samples, the 97.5th percentile from SW, and the 99th percentile from NW for adult men were higher than the PMTDI. The 90th percentile of daily DON intake through wheat-based products from MLYV, YHV, NE, total samples, and the 99th percentile from NW and SW for adult women were higher than the PMTDI. These results indicate that the MOS values were more than 1 and increased health risks might exist for the group if they ingest highly contaminated wheat. Other publications reported similar results. The 75th percentile of DON intakes through wheat flour for Chinese children and adults, as estimated from various online sources, are 6.5 and 2.2 μg/kg bw/day [49], respectively. The highest estimated daily DON exposure values in Europe (1.04 μg/kg bw/day), as calculated from urine samples, were reported for workers in the production area of a Portuguese dough company (*n* = 21) [57].

In conclusion, the risk of DON intake through wheat-based products from MLYV is higher than that from the four other areas, and children showed higher levels of dietary DON exposure than adults. These results are similar to our point evaluation analysis.

## 4. Conclusions

We performed a quantitative cumulative health risk assessment of DON intake through wheat-based products from Chinese five main production areas for seven- to 10-year-old children and adults in Shanghai by the point evaluation and the probabilistic evaluation based on Monte Carlo simulation. The results showed the mean dietary DON intake was lower than the PMTDI. Children showed higher dietary DON exposure values than adults. However, the wheat-based products from MLYV posed higher health risk compared to that from the other areas. In addition, the 99th percentiles of dietary DON exposures exceeded the PMTDI, suggesting adverse health effects might occur if the consumers ingest highly contaminated wheat-based products. Furthermore, we would like to make the following recommendations: (1) Wheat from high-contamination production areas should be routinely monitored, and control programs of agricultural practices should be set up, and (2) children and adults should consume wheat from NE, NW, or SW to avoid health risks associated with excessive DON intake. An additional study on seniors is warranted.

## Figures and Tables

**Table 1:** The daily intake of wheat-based products for inhabitants in Shanghai (g/d).

Group	Mean ± SD	Median	90th Percentile	95th Percentile	97.5th Percentile	99th Percentile
Boys ^1^	98.7 ± 120.5	67.0	230.0	303.4	361.5	513.6
Girls ^2^	75.1 ± 83.8	57.9	181.1	257.9	285.5	309.4
Adult men ^3^	95.5 ± 111.8	67.0	250.0	313.3	401.7	488.4
Adult women ^4^	72.2 ± 98.2	40.0	200.0	257.0	328.5	427.2

^1^ The mean weight of 7- to 10-year-old boys is 38.3 kg; ^2^ The mean weight of 7- to 10-year-old girls is 33.4 kg; ^3^ The mean weight of adult men is 62.7 kg; ^4^ The mean weight of adult women is 54.0 kg.

**Table 2:** DON contamination level in wheat-based products in Shanghai (µg/kg).

Geographic Origin of the Wheat	Number of Samples (*n*)	Number of Positivity (%)	Range of DON in Positivity Samples	Mean ± SD ^1^	Median	90th Percentile	95th Percentile	97.5th Percentile	99th Percentile
MLYV	122	103 (84.4)	41.8–1110	288.2 ± 269.6	202.5	756	850.9	924.6	972.9
YHV	140	117 (83.6)	42.0–896	148.7 ± 188.8	92.3	350.4	533.4	860.5	886.3
NE	25	25 (100)	43.3–297	137.4 ± 88.1	132.6	235.4	266.2	281.7	290.9
NW	25	20 (80.0)	46.6–131	66.7 ± 46.4	67.4	112	121.4	126.1	128.9
SW	28	23 (82.1)	48.2–120	70.4 ± 42.3	89.3	107.6	113.7	116.7	118.6
Total	340	288 (84.7)	41.8–1110	205.6 ± 234.7	129.4	500.6	840.2	879.1	934.2

^1^ The value of limit of detection (LOD) was assigned to all results below the LOD.

**Table 3:** T The estimated dietary DON exposure through wheat-based products from different areas for 7- to 10-year-old boys determined by point evaluation (µg/kg bw/day).

Geographic Origin of the Wheat	Mean	Median	90th Percentile	95th Percentile	97.5th Percentile	99th Percentile
MLYV	0.743	0.355	4.55	6.75	8.74	13.1
YHV	0.383	0.162	2.11	4.23	8.13	11.9
NE	0.354	0.232	1.42	2.11	2.66	3.90
NW	0.172	0.118	0.673	0.963	1.19	1.73
SW	0.182	0.156	0.647	0.902	1.10	1.59
Total	0.530	0.227	3.01	6.66	8.31	12.5

**Table 4:** The estimated dietary DON exposure through wheat-based products from different areas for 7- to 10-year-old girls determined by point evaluation (µg/kg bw/day).

Geographic Origin of the Wheat	Mean	Median	90th Percentile	95th Percentile	97.5th Percentile	99th Percentile
MLYV	0.648	0.351	4.10	6.57	7.90	9.01
YHV	0.334	0.160	1.90	4.12	7.35	8.21
NE	0.309	0.230	1.28	2.06	2.41	2.69
NW	0.150	0.117	0.607	0.937	1.08	1.19
SW	0.158	0.155	0.583	0.878	0.997	1.10
Total	0.462	0.224	2.71	6.49	7.51	8.65

**Table 5:** The estimated dietary DON exposure through wheat-based products from different areas for adult men determined by point evaluation (µg/kg bw/day).

Geographic Origin of the Wheat	Mean	Median	90th Percentile	95th Percentile	97.5th Percentile	99th Percentile
MLYV	0.439	0.216	3.01	4.25	5.95	7.58
YHV	0.226	0.099	1.40	2.67	5.51	6.90
NE	0.209	0.142	0.939	1.33	1.81	2.27
NW	0.102	0.072	0.447	0.607	0.808	1.01
SW	0.107	0.095	0.429	0.568	0.748	0.924
Total	0.313	0.138	1.99	4.20	5.63	7.28

**Table 6:** The estimated dietary DON exposure through wheat-based products from different areas for adult women determined by point evaluation (µg/kg bw/day).

Geographic Origin of the Wheat	Mean	Median	90th Percentile	95th Percentile	97.5th Percentile	99th Percentile
MLYV	0.385	0.150	2.80	4.05	5.63	7.70
YHV	0.199	0.068	1.30	2.54	5.24	7.01
NE	0.184	0.098	0.872	1.27	1.71	2.30
NW	0.089	0.050	0.415	0.578	0.767	1.020
SW	0.094	0.066	0.399	0.541	0.710	0.938
Total	0.275	0.096	1.85	3.99	5.348	7.39

## Data Availability

Not applicable.
